# Use of antibiotic cement-impregnated intramedullary nail in treatment of infected non-union of long bones

**DOI:** 10.4103/0019-5413.55468

**Published:** 2009

**Authors:** Ashok K Shyam, Parag K Sancheti, Salim K Patel, Steve Rocha, Chetan Pradhan, Atul Patil

**Affiliations:** Sancheti Institute for Orthopaedics and Rehabilitation, 16, Shivajinagar, Pune - 5, India

**Keywords:** Antibiotic cement-impregnated intramedullary nail, bone defect, infected non-union

## Abstract

**Background:**

In cases with infected non-union, the primary step is eradication of the infection before attempting to achieve union. Release of antibiotics from the bone cement at a high concentration and its penetration to the surrounding tissues, including cortical and cancellous bone, prompted the use of antibiotic cement in the control of bone infection. The aim of this study is to summarize our experience with the use of antibiotic cement-impregnated intramedullary nail (ACIIN) for control of infection in cases of infected non-union with bone defect.

**Materials and Methods:**

We prospectively studied 25 cases of infected non-union (23 femora and two tibiae). There were 24 males and one female, with the mean age being 33 years (range, 21–58 years). All patients had high-velocity road traffic accidents except one patient who had farmland injury. There were seven closed fractures, one grade I compound, two grade II compound fractures, five grade IIIA compound fractures, and 10 grade IIIB compound fractures. ACIIN was used in all cases after adequate debridement. Patients were classified according to the amount of bone defect present after debridement: group 1 with bone defect <4 cm (n=13), group 2 with bone defect ≥4–<6 cm (n=7), and group 3 with bone defect ≥6 cm (n=5). Infection control was judged on the basis of discharge through the wound and laboratory parameters. All patients were followed-up, with an average follow-up time of 29 months (range, 18–40 months). The mean duration of retention of the intramedullary rod was 8 weeks (range, 6–12 weeks).

**Results:**

In group 1, all cases achieved infection control, with three patients achieving bone union without any need of secondary procedure. In group 2, all cases achieved infection control but the time taken was significantly longer than for group 1 (*P* value 0.0002). All the cases required a secondary procedure in the form of either interlocking intramedullary nailing with iliac crest bone graft or Ilizarov ring fixator application to achieve union. None of the cases in group 3 achieved infection control.

**Conclusion:**

ACIINs are useful for infection control in cases of infected non-union with bone defect <6 cm. In cases with defect >6 cm, other alternatives should be used.

## INTRODUCTION

Infected non-union of long bones is a chronic and debilitating disorder that still poses a very complex problem to the surgeon today in terms of cost- and time-effective treatment.^1^ Causes of infected non-union are generally inherent to the fracture, like compound wound, loss of soft tissue or bone, severe comminution, and gross displacement.^2^ Insufficient immobilization, bone defect, and infection are cited as the main causes of persistent non-union.^2^ Traditionally, treatment of non-union follows a two-stage procedure. The first stage comprises of debridement with or without antibiotic cement bead insertion and systemic antibiotics to convert an infected non-union to a aseptic non-union. The second stage is performed to achieve stability either by external or internal fixation and bone grafting.^2^–^7^ Single-staged procedures such as debridement and application of Ilizarov fixator^1^,^8^–^12^ or use of antibiotic cement-impregnated intramedullary nails (ACIINs)^13^–^16^ have been described in the literature. Ilizarov ring fixator has been used after debridement for bone transport or corticotomy distraction after acute docking. This procedure is technically demanding and has significant complications and is best suited for large segmental bone defects.^1^,^8^–^12^ Several authors have promoted ACIIN as a simple, inexpensive, and very effective single-stage procedure for treating infected non-union. The ACIIN fills in the dead space, provides a high concentration of antibiotics locally, and gives good mechanical stability.^13^–^16^ However; these studies have used ACIIN only in cases without significant bone defect. The present study was undertaken to analyze the usefulness of the ACIIN in cases of infected non-union with bone defects.

## MATERIALS AND METHODS

Twenty-five consecutive patients presenting to our institute with infected non-union were enrolled for the prospective study during 2004–2006 [Table 1]. There were 24 males and one female, with the mean age being 33 years (range, 21–58 years). Twenty-three femora and two tibias were treated, with 24 patients having sustained high-velocity trauma and one patient having sustained farmland injury. All patients had established infected non-unions, with 17 patients undergoing two or more procedures (average, 3.2 procedures) like implant removal, repeated debridement, external fixator application, and upper tibial pin traction that failed to achieve union. There were seven closed fractures, one grade I compound, two grade II compound fractures, five grade IIIA compound fractures, and 10 grade IIIB compound fractures.^17^ Twenty-one patients had intramedullary nail *in situ* when they presented, three patients had external fixator, and one patient had Ilizarov ring fixator *in situ*. The mean duration of infection was 7.7 months (range, 3–14 months). All the patients had draining sinuses, with 17 patients having cultures positive for *Staphylococcus aureus*. The remaining were culture negative. Sinograms were performed in cases with multiple sinuses to delineate their pathoanatomy. All patients were receiving antibiotics at the time of culture. Before surgery, antibiotics according to the sensitivity reports were started for the culture-positive cases and the remaining were started on broad spectrum antibiotics, namely second- or third-generation cephalosporin and gentamicin. Only one patient was diabetic, which was adequately controlled on insulin.

**Table 1:** Pre operative details of the cases of infected nonunion of long bones of lower extremity

Patient no.	Age/sex	Bone	Classification^a^	Initial treatment	Duration of infection (months)
Group 1 (bone defect <4 cm)					
1	58/M	Femur	Closed	ILN	4
2	45/M	Femur	II	Deb, Ilizarov fixator	9
3	33/M	Tibia	I	Deb, ILN	5
4	29/M	Femur	IIIA	Deb, UTPT, ILN	8
5	44/M	Femur	IIIB	Deb, Ex-fix	7
6	35/M	Femur	IIIB	Deb, ILN	9
7	26/M	Tibia	Closed	ILN	7
8	38/M	Femur	Closed	ILN	5
9	32/M	Femur	IIIB	Deb, UTPT, ILN	8
10	25/M	Femur	Closed	ILN	3
11	29/M	Femur	Closed	ILN	3
12	30/M	Femur	Closed	ILN	7
13	25/M	Femur	IIIB	Deb, UTPT, ILN	7
Group 2 (bone defect ≥4–<6 cm)					
14	36/M	Femur	IIIA	Deb, ILN	6
15	35/M	Femur	II	Deb, UTPT, ILN	6
16	24/F	Femur	IIIA	Deb, Ex-fix	10
17	23/M	Femur	IIIA	Deb, UTPT, ILN	9
18	35/M	Femur	IIIA	Deb, ILN	7
19	28/M	Femur	IIIB	Deb, UTPT, ILN	13
20	22/M	Femur	IIIB	Deb, ILN	10
Group 3 (bone defect ≥6 cm)					
21	21/M	Femur	IIIB	Deb, Ex-fix, ILN	10
22	42/M	Femur	IIIB	Deb, Ex-fix	9
23	47/M	Femur	IIIB	Deb, UTPT, ILN	11
24	34/M	Femur	IIIB	Deb, Ex-fix, ILN	14
25	25/M	Femur	Closed	ILN, Deb	6

a = Classification according to the Gustilo Anderson classification, Deb = Debridement, ILN = Intramedullary interlock nailing, Ex-fix = External fixator, UTPT = Upper tibial pin traction.

### Operative procedure

**Figure 1:**
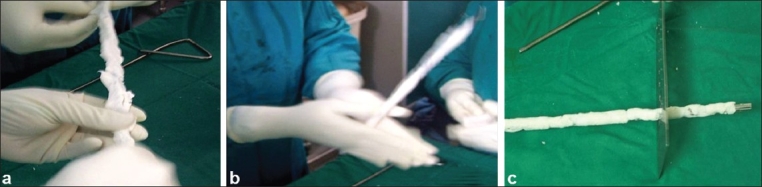
Photograph showing the method of application of cement to the intramedullary rod (a, b). Size of the antibiotic-impregnated intramedullary nail is checked with a sizer (c)

Implant removal was performed first. The sinus tracts were injected with methylene blue and were excised till the bone. The fracture site was opened and radical debridement was performed with excision of the infected bone ends, scarred soft tissue, and granulation tissue. The intramedullary canal was reamed to size 2 mm more than the previous nail or till the fresh bleeding bone was reached. The reamings and granulation tissue were sent for culture. The wound and the entire medullary canal were cleaned with pulse lavage of about 4–5 L of normal saline. The antibiotic-impregnated cement rod was prepared using K-nail for femur and V-nail for tibia. The recommended mix ratio of antibiotic to bone cement is 25 ml of antibiotic to a pack of 40 g cement.^18^ A higher mix ratio will alter the setting time markedly as also the handling properties. We used 2 g of vancomycin and 2 g of gentamicin mixed with every 40 g of bone cement. An intramedullary nail of size 2 mm less than the largest reamer was used, with a 6 or 7 mm nail being used in most of the cases. Manual mixing of cement was performed and the cement was applied to the nail in a uniform fashion [Figure 1a–b]. The eye of the nail was kept uncemented to facilitate easy removal. Size of the nail was checked using a nail width measuring scale [Figure 1c]. Uniform thickness was achieved by repeatedly passing it through the width measuring scale. A fairly smooth and even surface is obtained by manual rolling with repeated checking of the width by the scale. The nail was kept in air for 15 min to allow for evaporation of the monomer. Retrograde insertion of the nail is performed through the fracture site for femur cases and antegrade insertion of the nail was performed for tibia cases. In cases where resistance was encountered during insertion, a further reaming was performed. In cases with bone defects, an attempt to approximate the bony ends was made. The wound was again washed thoroughly and closure was performed without drain.

Anteroposterior, lateral, and oblique radiographs were taken at the time of admission into the study and thereafter following procedures involving fixation/stabilization and at regular intervals of 4 weeks. A complete blood count (CBC), erythrocyte sedimentation rate (ESR), and C-reactive protein (CRP) levels were performed initially and then at regular biweekly intervals to record rising or falling trends. All patients were administered 4–6 weeks of intravenous antibiotics according to the culture reports. Infection control was judged on the basis of discharge from the wound, clinical signs of inflammation, and laboratory parameters like CBC, ESR, and CRP.

Patients were classified according to the amount of bone defect present after radical debridement. The bone defect was measured after debridement by applying manual tension to the soft tissues on the table under anesthesia; group 1 with bone defect <4 cm (n=13), group 2 with bone defect ≥4–< 6 cm (n=7), and group 3 with bone defect ≥6 cm (n=5). The three groups were compared with respect to control of infection by the primary procedure, requirement of secondary procedure, and bony union. The duration of infection control after surgery was compared between the groups and significance was checked using paired t-test, with a *P* value <0.05 being considered significant.

## RESULTS

The intraoperative specimen reported positive culture for all 25 cases including the five culture-negative cases pre-operatively. Twenty-two samples were positive for *Staphylococcus aureus*, two samples were positive for *Pseudomonas aeruginosa*, and one sample was positive for *Klebsiella*. All patients were followed-up, with an average follow-up time of 29 months (range, 18–40 months). The mean duration of retention of the intramedullary rod was 8 weeks (range, 6–12 weeks).

**Figure 2:**
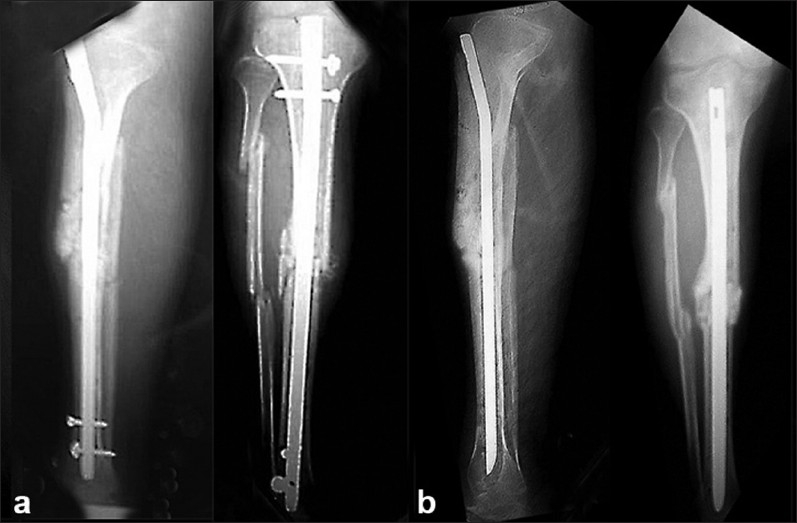
Lateral and anteroposterior (a) X-ray of a closed fracture middle 1/3rd tibia treated with conventional interlocking nail, which subsequently developed infection. Union was achieved with debridement and antibiotic cement-impregnated intramedullary nail and a radiograph taken at 18 months follow-up (b)

There were 13 patients in group 1, with an average defect of 2.2 cm (range, 0–3.2 cm). All the patients achieved infection control with normal laboratory parameters and no active discharge in an average of 6.1 weeks (range, 4–8 weeks). Two cases in this group that had no bone defect after debridement and one case with a 2-cm bone defect attained union with the cemented nail *in situ* [Figure 2] in 7, 13, and 8 months, respectively. The remaining patients in this group underwent nail removal followed by interlocking nail with bone grafting at the fracture site. Cultures obtained at the time of rod removal were negative in all patients. Fracture union was seen in all the cases after the secondary procedure [Table 2], with the average time to union being 14 months (range, 7–18 months) for this group.

**Table 2:** Post-operative details of the patients in the study

Patient no.	Bone defect (cm)	Infection control	Duration of infection control (weeks)	Secondary procedure	Union
Group 1 (bone defect <4 cm)					
1	2	Controlled	4	None	Yes
2	3	Controlled	5	ILN+BG	Yes
3	0	Controlled	4	None	Yes
4	3	Controlled	7	ILN+BG	Yes
5	2	Controlled	8	ILN	Yes
6	3	Controlled	8	ILN+BG	Yes
7	0	Controlled	7	None	Yes
8	3	Controlled	5	ILN+BG	Yes
9	3	Controlled	8	ILN+BG	Yes
10	2	Controlled	7	ILN	Yes
11	2.5	Controlled	6	ILN+BG	Yes
12	3	Controlled	5	ILN+BG	Yes
13	3.2	Controlled	6	ILN+BG	Yes
Group 2 (bone defect ≥4–<6 cm)					
14	4	Controlled	7	ILN+BG	Yes
15	4.4	Controlled	8	ILN+BG	Yes
16	5	Controlled	6	Ilizarov fixator	Yes
17	4.5	Controlled	10	ILN+BG	Yes
18	4	Controlled	11	ILN+BG	Yes
19	5.1	Controlled	9	Ilizarov fixator	Yes
20	4	Controlled	9	ILN+BG	Yes
Group 3 (bone defect ≥6 cm)					
21	6.2	Uncontrolled	-	Deb, Ilizarov fixator	Yes
22	7	Uncontrolled	-	Deb, Ilizarov fixator	Yes
23	6.3	Uncontrolled	-	Deb, Ilizarov fixator	Yes
24	7.5	Uncontrolled	-	Deb, Ilizarov fixator	No
25	6	Uncontrolled	-	Deb, Ilizarov fixator	Yes

Deb = Debridement, ILN = Intramedullary interlock nailing, Ex-fix = External fixator, UTPT = Upper tibial pin traction

**Figure 3:**
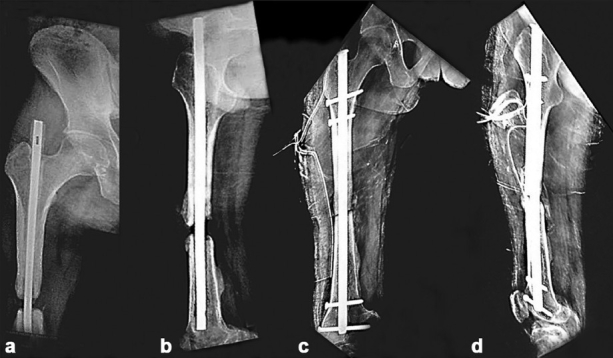
Anteroposterior (a) and lateral (b) radiograph of right thigh including hip depicting a gap non-union of femur with infection treated with antibiotic impregnated intramedullary nail. Anteroposterior (c) and lateral (d) radiograph of the same patient showing exchange interlock nailing performed after control of infection

There were seven patients in group 2 with a mean bone defect of 4.4 cm (range, 4–5.1 cm). All patients achieved infection control in mean of 8.6 weeks (range, 6–11 weeks). All the cases required a secondary procedure to achieve union. When shortening was <3 cm, exchange nailing and autogeous corticocancellous iliac crest bone grafting (n=5) were performed [Figure 3] and when shortening of more than 3 cm was noted, an Ilizarov ring fixator was applied and leg lengthening was performed (n=2). All the patients achieved union after the secondary procedure [Table 2], with the average time to union being 14.5 months (range, 12–18 months) for this group. Control of infection took a significantly longer time for patients in group 2 as compared with group 1 (*P* value 0.0002) [Table 3].

**Table 3:** Comparison of the duration of infection control between group 1 and group 2

Variable	Group 1	Group 2	*P* value
No. of patients	13	7	
Duration of infection control (mean+SD)	6.15±1.46	8.57±1.72	0.0002

*P* value is for the paired t-test. Duration of infection is in weeks.

In group 3, there were five patients with an average bone defect of 6.6 cm (range, 6–7.5 cm). All the patients had persistent infection even after 12 weeks of antibiotic impregnated intramedullary nailing. They all had intermittent draining sinuses with raised laboratory parameters. These patients underwent a secondary debridement with nail removal and application of Ilizarov ring fixator. Corticotomy and acute docking were performed with gradual lengthening at the corticotomy site. Union was achieved by this procedure in four cases [Table 2], with the average time to union being 17.4 months (range, 16–20 months). In one case, the infection became quiescent and a stiff non-union was achieved at the fracture site. This patient refused further surgery and was mobilized with a functional femoral brace.

Twenty-two patients required a secondary procedure during the course of their treatment. Sixty-eight percent of the patients (n=17) had poor skin conditions due to multiple surgical procedures, healed sinuses and scars leading to cicaterization. Thirty-two percent of the patients (n=8) had a knee range <60°. Eighty-eight percent of the patients (n=22) had passive hip flexion only up to 90°. However, all the patients were able to walk full-weight bearing and pursue their daily activities. At an average follow-up time of 29 months (range, 18–40 months), there was no recurrence of infection.

## DISCUSSION

Thorough debridement, rigid fixation, and prolonged antibiotics are the mainstay in treatment of infected non-union of the long bone.^1^–^2^,^19^–^21^ A variety of staged procedures have been described for the management of infected non-union. Intramedullary devices have been used in both primary stage of infection control^13^–^16^ and in secondary stage of bone healing^22^ with good results. Bone defect following debridement increases the complexity of the management.^23^ We studied the appropriateness of the use of ACIIN in cases of infected non-union with bone defect.

The infected foci within the bone are surrounded by a sclerotic, relatively avascular bone covered by a thickened periosteum, scarred muscle and subcutaneous tissue. This avascular envelope of scar tissue leaves systemic antibiotics essentially ineffective. This explains the positive cultures in a majority of our patients even though all patients were receiving broad spectrum antibiotics at the time of sinus tract culture. However, few culture reports were negative in which case either the organism is highly sensitive to the antibiotics or infection is by a fastidious organism like hemolytic *Streptococci* or *Enterococci*. In former situations, the dead bone acts as a foreign body and continues to generate discharge from the wound. Negative sinus tract culture reports have been reported by other researchers too who reported it to have a very low sensitivity, specificity, and positive predictive value.^24^–^26^ We found all the intraoperative specimen cultures to be positive, suggesting it to be a more preferable method to decide an appropriate antibiotic regimen in chronic osteomyelitis.

Intramedullary infection leading to non-union of the fracture is a known complication of intramedullary nailing.^27^ Compound fractures have a higher incidence than closed fractures treated with intramedullary nailing.^28^,^29^ Our series had seven closed fractures and 18 compound fractures, of which 23 were treated with intramedullary nailing thus concurring with previous reports. Classifications of infected non-union with bone defect are based on the ease of reconstruction of the bone defect after the infection is settled. May *et al*.^30^ classified bone defect into <6 cm and >6 cm, while Jain and Sinha^19^ proposed a classification of defect into <4 cm and >4 cm. We combined the two classifications to make our three groups so as to compare our results.

Use of antibiotic-impregnated cement was first noted by Buchholz and Engelbrecht.^31^,^32^ A high local concentration of antibiotics and low systemic side effects were the major advantage.^33^,^34^ Gentamicin has been the most widely used agent followed by vancomycin.^32^–^35^ Use of two antibiotics, namely gentamicin and vancomycin, with bone cement widens the spectrum of activity and also enhances the elution properties of the two antibiotics.^35^,^36^ Klemm was the first to use antibiotic cement beads in cases of osteomyelitis.^37^ Cement beads fill the dead space and also allow a high concentration of local antibiotics. The effectiveness of the antibiotic-impregnated cement beads in the control of bone infection is well established. Cement beads have been used for intramedullary infections. However, they offer no mechanical stability and are difficult to remove after 2 weeks.^38^ ACIIN can provide stability, is easy to remove, and also provides all the advantages of the cement beads. Use of ACIIN has been first reported by Paley and Herzenberg^13^ and later by other authors.^14^–^16^,^39^ Only one study by Thonse and Conway has studied cases of infected non-union with bone defects in 20 patients. They were able to achieve primary union by primary use of ACIIN in only two cases with bone defect, with the remaining cases requiring secondary procedure. They reported infection control in 95% of their cases (n=19). In our study, only three patients achieved primary bone union with the use of ACIIN, of which two patients did not have any bone defect and one patient had a bone defect of 2 cm [Table 2]. In group 2, none of the patients achieved primary bone union and a significantly longer time was required for infection control when compared with group1. In group 3, the goal of infection control was not achieved in any of the cases and all required secondary procedures for infection control and bony union.

## CONCLUSION

We find that ACIIN was a good procedure to achieve early primary infection control in cases of infected non-union with bone defect <4 cm. ACIIN is useful for infection control in cases with bone defects from 4 to 6 cm; however, it takes a significantly longer time when compared with patients with lesser bone defect. It should not be used in cases with bone defects >6 cm, where it fails to achieve an adequate stability and infection control, and for these cases procedures like Ilizarov fixator with bone transport or lengthening with acute docking should be used.
